# A Scalable Bacterial Cellulose Ionogel for Multisensory Electronic Skin

**DOI:** 10.34133/2022/9814767

**Published:** 2022-06-02

**Authors:** Geyuan Jiang, Gang Wang, Ying Zhu, Wanke Cheng, Kaiyue Cao, Guangwen Xu, Dawei Zhao, Haipeng Yu

**Affiliations:** ^1^Key Laboratory on Resources Chemicals and Materials of Ministry of Education, Shenyang University of Chemical Technology, Shenyang 110142, China; ^2^Key Laboratory of Bio-Based Material Science and Technology of Ministry of Education, Northeast Forestry University, Harbin 150040, China; ^3^Tianjin Key Laboratory of Pulp and Paper, Tianjin University of Science and Technology, Tianjin 300457, China

## Abstract

Electronic skin (e-skin), a new generation of flexible electronics, has drawn interest in soft robotics, artificial intelligence, and biomedical devices. However, most existing e-skins involve complex preparation procedures and are characterized by single-sensing capability and insufficient scalability. Here, we report on a one-step strategy in which a thermionic source is used for the in situ molecularization of bacterial cellulose polymeric fibers into molecular chains, controllably constructing an ionogel with a scalable mode for e-skin. The synergistic effect of a molecular-scale hydrogen bond interweaving network and a nanoscale fiber skeleton confers a robust tensile strength (up to 7.8 MPa) and high ionic conductivity (up to 62.58 mS/cm) on the as-developed ionogel. Inspired by the tongue to engineer the perceptual patterns in this ionogel, we present a smart e-skin with the perfect combination of excellent ion transport and discriminability, showing six stimulating responses to pressure, touch, temperature, humidity, magnetic force, and even astringency. This study proposes a simple, efficient, controllable, and sustainable approach toward a low-carbon, versatile, and scalable e-skin design and structure–performance development.

## 1. Introduction

Flexible electronics, which are portable and practicable, have flourished in recent decades [1]. Electronic skin (e-skin), which is flexible, can transduce mechanical or physical stimulations into recognizable electronic data for analysis and readout [2–4], showing great potential application in robotics and bioelectronics [5–13]. In an attempt to realize degrees of softness and comfort close to those of the human skin, chemically synthesized polymer materials with flexibility and stretchability need to be developed via complex machine processing [14–16]. However, these polymer substrates with poor degradability are electronic insulators and possess no ionic conductivity; thus, some conductive materials need to be introduced into the polymers by mixing, layer-by-layer stacking, or three-dimensional printing to achieve signal capture and feedback [15–18]. These processes are undoubtedly tedious and carbon-intensive and lead to problems such as poor interface stability (between the conductive network and the flexible substrate) and environmental pollution.

As a polysaccharide polymer cellulose, bacterial cellulose (BC) is characterized by good biocompatibility, adaptability, and air permeability. As such, BC is often used as a wound dressing for human skin and tissue repair. A nanoscale BC fiber endows BC materials, such as hydrogels, with good flexibility and mechanical property (Figure S1). However, the internal microstructure of BC hydrogels is still not sufficiently delicate and lacks molecular-scale structure regulation and design. These qualities limit the development of functional BC-based gel materials and their application in e-skin. Utilizing ionic liquids or deep-eutectic solvents to break the hydrogen bonds between cellulose molecules, these interesting works based on hydrogen-bonding (H-bonding) topological network regulation and molecular self-assembly have been conducted on the preparation of cellulosic ionic materials [19–22]. For example, we used a green imidazole-based ionic liquid as solvent and reported a dynamic cellulosic ionic gel with a variable microstructure, which showed its feasibility for application in flexible, self-healing e-skin (with a good sensitivity to touch and humidity) [23]. However, despite its features of self-healing and skin-friendliness, this dynamic ionic gel involves complex fabrication steps, requires a large amount of energy, and shows poor scalability. Unlike human skin, this e-skin cannot sense temperature and pressure stimulation owing to a lack of heat- or force-sensitive factors.

**Figure 1:**
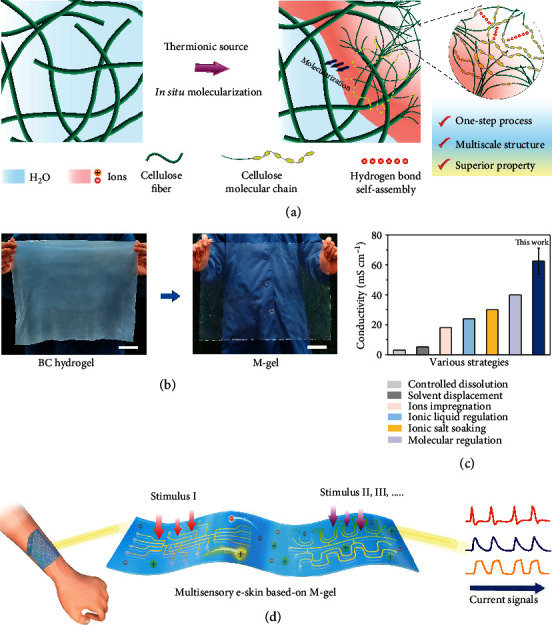
Design and construction of M-gel. (a) Schematic of the preparation of M-gel by using an in situ molecularization strategy. (b) Optical images of the BC hydrogel translated into M-gel for e-skin. Scale bar, 5 cm. (c) Comparison of ionic conductivity between M-gel and reported ionic materials. (d) Multisensory e-skin from M-gel exhibiting diverse stimulus responsiveness.

To achieve low-carbon and sustainable development, the design route needs to be simplified, the materials to be used should be green, and multifunctional panels have to be constructed for fabricating multisensory e-skin. Considering the aforementioned challenges, we propose an in situ molecularization strategy for introducing a thermionic liquid of 1-butyl-3-methylimidazolium chloride ([Bmim]Cl) into a monolithic BC hydrogel to convert cellulose fibers into molecular chains (Figure 1(a)). This facile and one-step strategy enables the scalable production of molecularized gel materials with both excellent ionic conductivity and high mechanical strength from resource-abundant biomass materials. By controlling the stimulation time of the thermionic source to coordinate the reaction–diffusion relationship between ions, water, and cellulose, this molecularized ionogel (called M-gel) owns a blended multiscale structure: a molecular-scale H-bonding topological network and a nanoscale fiber skeleton (Figure 1(a)). This structure endows the M-gel with good transparency (Figure 1(b)), flexibility, tunable mechanical performance, and high ionic conductivity reaching 62.58 mS/cm, which is superior to existing ionic gel materials (Figure 1(c)) [24–28]. In addition, the degree of molecularization (DM) of M-gel can be quantitatively designed and regulated to be between 0 and 100% by controlling the thermionic treatment time. Inspired by the distinct sensing structure of the human tongue, we developed an e-skin with a multisensory behavior by designing and integrating the respective perception patterns in this M-gel (Figure 1(d)). This e-skin device, as a proof-of-concept demonstration, showed ideal multistimulus responsiveness and recognizability to pressure, touch, temperature, humidity, magnetic force, and astringency (this stimulus is only perceived by the tongue).

## 2. Results and Discussion

### 2.1. Design, Construction, and Characterizations of the M-Gel

**Figure 2:**
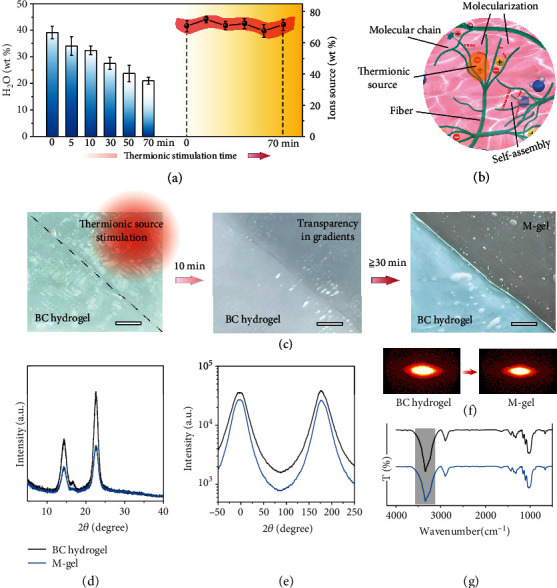
Molecularization and characteristics of the M-gel. (a) Tunable behavior of the H_2_O and ion contents during thermionic stimulation. (b) Schematic of the molecularization and self-assembly. (c) Real-time optical images of the molecularization. Scale bar, 1.5 cm. (d) XRD spectra of the BC hydrogel and M-gel. (e) Small-angle X-ray scattering (SAXS) patterns of the BC hydrogel and M-gel. (f) Azimuthal-integrated intensity distribution curves of the 2D SAXS patterns of the BC hydrogel and M-gel. (g) FTIR spectra of the BC hydrogel and M-gel.

As a cellulose material, the BC hydrogel possesses a nanoscale fiber structure (Figure S1) and has water content reaching 40 wt.%. Water molecules (H_2_O) show H-bonding with the hydroxyl of the BC fiber, conferring excellent flexibility and mechanical performance on the BC hydrogel (Figure S1). However, when the thermionic sources of [Bmim]^+^ and Cl^−^ are introduced into the BC hydrogel (Figure S2), the diffusion-driven Turing instability between cellulose, H_2_O, and ions occurs in the system [23]. On the one hand, the ions of [Bmim]^+^ and Cl^−^ (as the activator) prefer to interact with BC fibers and form H-bonding interactions with the hydroxyl protons of cellulose to destroy the intermolecular/intramolecular H-bonding network and thus obtain a molecular chain (referred to as a “molecularization”); meanwhile, H_2_O (as the inhibitor) tends to prevent this behavior by inducing the formation of H-bonding between cellulose molecules (referred to as the “self-assembly,” Figure 1(a)). By controlling the stimulation time, we can expediently adjust the content of H_2_O (from 39.16 wt.% to 21.01 wt.%) and ions (around 70 wt.%, Figure 2(a)), thus realizing the dynamic regulation of this competitive relationship between molecularization and self-assembly to design the structure and property of the M-gel material (Figure 2(b)).

To observe the effect of this in situ molecularization process, a comparative experiment was performed on a piece of BC hydrogel. The upper right part of the hydrogel was treated with the thermionic source, unlike the lower left part (Figure 2(c)). After heat treatment at 80°C for 10 min, the originally white and opaque BC hydrogel achieved slight transparency. For 30 min, a transparent M-gel appeared, with light transmittance exceeding 85% (Figure S3). However, the part of the BC hydrogel without thermionic source treatment showed no intuitive changes. Observation of the X-ray diffraction (XRD) spectrum revealed that the M-gel (treated with a thermionic source for 30 min) exhibited a sharp crystalline peak similar to that of the BC hydrogel (Figure 2(d)), indicating that this M-gel retained the ordered crystalline regions.

SAXS was performed to detect subtle changes in the topological network. As shown in Figure 2(e), the peaks from the M-gel appear sharper than those of the BC hydrogel; in Figure 2(f), the 2D SAXS scattering signal of the M-gel also exhibits a sharper pattern than that of the BC hydrogel. These results indicate that the M-gel had a molecular-scale topological network. Visualization by Fourier transform infrared spectroscopy (FTIR) (Figure 2(g)) reveals the sharp peak of the M-gel at 3400 cm^−1^, ascribed to the stretching vibration behavior of hydroxyl, which indicates the rich H-bonding network of the M-gel. These rich H-bonds, high crystallization, and molecular-scale topology network can undoubtedly endow the M-gel material with various beneficial properties.

### 2.2. Morphological Structure Design and Regulation

**Figure 3:**
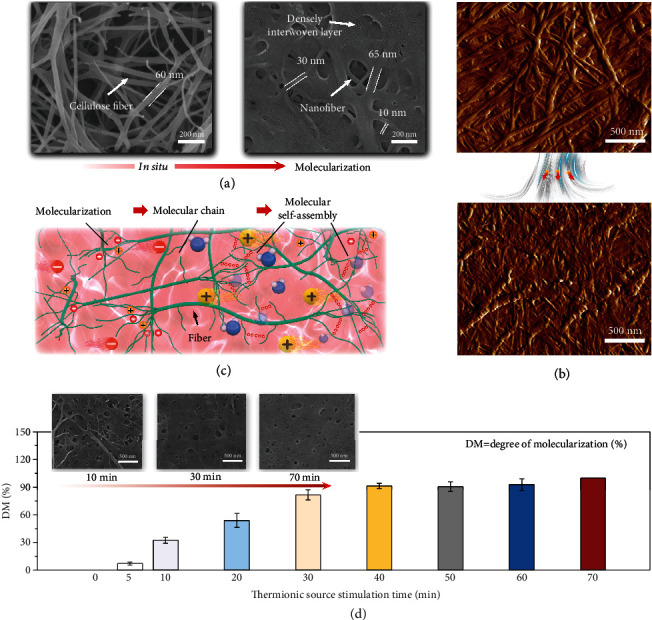
Morphological design and regulation of the M-gel. (a) SEM images of the BC hydrogel and the M-gel. (b) AFM images of the BC hydrogel and the M-gel. (c) Schematic diagram of the M-gel with the multiscale structure of both the molecular-scale H-bonding topology network and nanoscale fibers. (d) Controllable DM of the M-gel by determining the reasonable thermionic treatment time. Insets show the real-time SEM images of the M-gel during different periods of thermionic molecularization.

To better demonstrate the multiscale structure of the M-gel, analytical procedures, such as scanning electron microscopy (SEM) and atomic force microscopy (AFM), were conducted to examine the morphology and microstructure of the M-gel. Compared with the BC hydrogel consisting of only cellulose fibers, the M-gel treated via molecularization showed an evident multiscale structural feature, with both a dense interwoven layer and a nanoscale fiber with different diameters (Figure 3(a)). Using AFM and images, we also examined this densely layered structure, which was flawlessly embedded in the nanoscale fiber skeleton, forming a seamless whole (Figure 3(b)). This combination can confer strong mechanical properties and performance durability on the M-gel material. This distinct morphological structure in the M-gel was derived from the competitive behavior between molecularization (turning fibers into molecular chains) and cellulose-molecule self-assembly (constructing the intermolecular H-bonding topological network, Figure 3(c)).

The thermionic source changed the original and rigid situation between cellulose and H_2_O, prompting the system to exhibit dynamic and tunable behavior patterns and performances. Notably, we can design the morphology and microstructure of the M-gel from a multiscale-coexisting body (both molecular self-assembly and nanofibers) to a fully interwoven dense structure by controlling the molecularization time of the thermionic source (insets of Figure 3(d)). Meanwhile, by calculating and analyzing the change in the light transmittance value (T %, Figure S3) of the M-gel over different molecularization periods, we can quantify and design the degree of molecularization (DM) of the M-gel. As shown in Figure 3(d), when treated with the thermionic source for 5, 10, 30, 50, and 70 min, the DM of M-gel can be controlled at 7.08%, 32.38%, 81.52%, 90.5%, and around 100%, respectively. In addition, the crystallinity of M-gel also exhibits a designability between 85% and 50.4% (Figure S4). So, this manipulation is controllable, customizable, and scalable; in addition, it is not only applicable to the BC hydrogel but can also be extended to other cellulosic materials such as filter paper and printing paper (Figure S5). To our knowledge, this structural design strategy for the ionogel has not been reported in the literature.

### 2.3. Excellent Mechanical Performance and Ionic Conductivity

**Figure 4:**
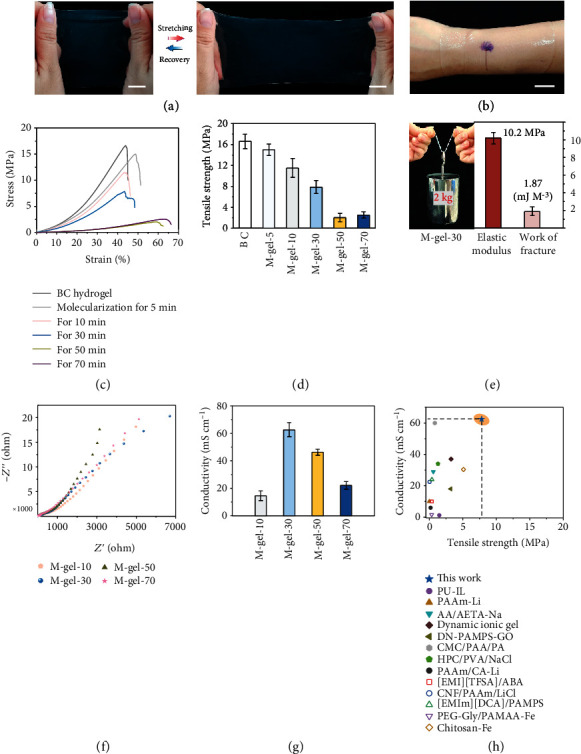
Mechanical properties and ionic conductivity of the M-gel. (a) M-gel with good switchability between stretching and recovery states. Scale bar, 1 cm. (b) M-gel closely attached to human wrists and exhibiting good transparency. Scale bar, 2 cm. (c) Tensile stress–strain curves for the M-gel treated over different lengths of molecularization time. (d) Comparison of the tensile strengths of the M-gel treated over different lengths of molecularization time. (e) Elastic modulus and work of fracture of M-gel-30. Optical image of M-gel-30 lifting 2 kg in weight. (f) Electrochemical impedance spectroscopy curves of M-gels treated over different lengths of molecularization time. (g) Comparison of the ionic conductivity of M-gels treated over different lengths of molecularization time. (h) Comparison of the mechanical and electronic performances between the proposed M-gel and other various ionic gel materials.

The M-gel presented excellent flexibility, transparency, and stretchability (Figure 4(a)), showing free and perfect switchability between stretching and recovery states (Figure S6). This M-gel can also be closely attached to human wrists and fingers under various large bending strains (Figure 4(b) and Figure S7). For an ionic gel material used in e-skin, biocompatibility (for human body), self-healing, and noncorrosiveness (for artificial limb) are important performance indicators. As shown in Figure S6 and S8, our M-gel shows good skin-friendliness (without tissue damage and inflammation when adhered to the human wrists for more than 10 h) and rapid self-healing (80°C for 30 min) and is not invasive to an artificial hand. On the basis of the tensile stress–strain tests (Figure 4(c)), the mechanical properties of the M-gel can be regulated, from a strength of 15 MPa (high) to 2.5 MPa. After molecularization for 30 min, the M-gel (referred to as M-gel-30) had a tensile strength of up to 7.8 MPa (Figure 4(d)), an elastic modulus of up to 10.2 MPa, and work of fracture of about 1.87 mJ m^−3^ (Figure 4(e)); these properties were superior to those of some high-performance cellulosic ionic gels [22, 23]. Meanwhile, M-gel-30 with robust toughness could easily lift a weight of 2 kg (over 50,000 times its weight, Figure 4(e)). Even with complete molecularization after thermionic treatment for 70 min, the M-gel still achieved a perfect tensile strength of 2.5 MPa, similar to that of human cartilage, and thus exhibited potential for application in artificial tissue for robots.

The introduction of the thermionic source endowed the M-gel with an ion-rich environment, which possessed programmable ionic conductivity. On the basis of the electrochemical impedance spectroscopy (EIS) curves in Figure 4(f), we calculated the ionic conductive behavior of the M-gel during different molecularization processes. As shown in Figure 4(g), the M-gel-30 achieves the highest ionic conductivity reaching 62.58 mS/cm, which is largely attributed to its multiscale structures. First, the nanoscale fiber skeleton provided a smooth linear path for transporting ions. Second, the molecular scale H-bonding topology network conferred abundant electrostatic-attraction sites for accelerating ion diffusion. Owing to excellent ionic conductivity, M-gel-30 as a flexible conductor can easily illuminate a light-emitting diode (LED) and shows satisfactory antistrain performance (Figure S9). In addition, M-gel-30 presented good conductive stability in an open environment with a relative humidity of ≈45% for more than 30 d (Figure S10). Even after 50 cyclic folding at 180°, the M-gel-30 still presents the high conductivity of 57 mS/cm and structural stability without any breakage and damage (Figure S11). To further demonstrate the excellent mechanical and electronic properties, we compared this M-gel with numerous ionic gel materials and found that both properties were superior (Figure 4(h)) [26–38].

### 2.4. Applications of the M-Gel in Constructing Multisensory e-Skin

**Figure 5:**
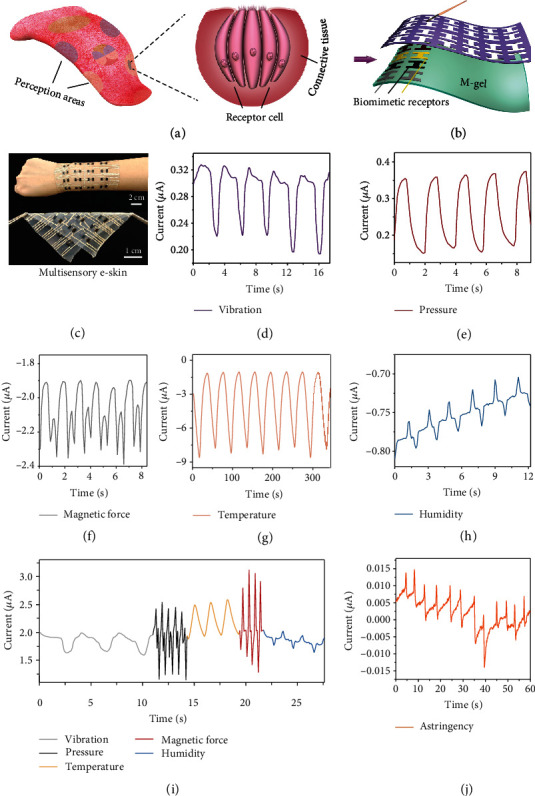
Construction of the multisensory e-skin. (a) Multisensory structure of the human tongue. (b) Fabrication of the e-skin via a simple brushing process on the M-gel. (c) Optical images of multisensory e-skin with good flexibility and adhesion to human wrists. Current waveforms of the e-skin sensing the (d) vibration, (e) pressure, (f) magnetic force, (g) temperature, and (h) humidity. (i) Multisensory e-skin simultaneously sensing the multistimuli. (j) Current waveforms of multisensory e-skin sensing the astringent stimuli from citric acid.

The human tongue as a soft and sensitive organ is highly capable of simultaneously sensing various taste stimuli (sour, sweet, bitter, spicy, and astringent) [39]. This function is attributed to the tongue possessing diverse perception areas (consisting of receptor cells and connective tissue, Figure 5(a)). Receptor cells buried in connective tissue have a regional structure and perception, which can distinguish the external stimuli and convert them into corresponding ion pulses. The connective tissue as a flexible conductor then transmits these ion signals to the nerve center of the human body, producing accurate behavioral feedback.

The proposed M-gel exhibits good flexibility, high ionic conductivity, and performance stability. It has a superior capability to transport ions, similar to that of the connective tissue of the tongue. In addition, owing to its rich active hydroxyl groups freed by molecularization, the M-gel showed an outstanding adhesive performance with strengths of up to 3.93 N (Figure S12). Inspired by the structure-feature of the tongue, we innovatively used our ionic conductive M-gel to mimic the connective tissue of the tongue while selecting the sensing materials of poly(3,4-ethylenedioxythiophene):poly(styrene sulfonate) (PEDOT:PSS, sensing temperature) [40], carbon nanotubes (CNTs, sensing pressure and deformation), Ag nanofiber (AgNWs, sensing deformation) [40], ionic gel (sensing humidity) [23], and nanonickel powder (sensing magnetism and temperature) to mimic the receptor cells (Figure S13). We successfully developed a flexible, transparent, and multisensory e-skin device on the basis of a simple brushing process in Figure 5(b). Notably, this e-skin was easily formed into large sizes, molded into diverse shapes, or imparted with various stimulation receptors, depending on the demand.

As shown in Figure 5(c), this multisensory e-skin exhibits adequate flexibility and seamless interface adhesion to human wrists. Moreover, this e-skin showed excellent structural integrity and stability even after several contacting abrasion and water immersion experiments (Figure S14) because of the strong H-bonding and coordination behavior between cellulose molecules and nanomaterials. Owing to its high ion conductivity and structural superiority, the e-skin, as a flexible electronic, presented ideal signal feedback to multistimulations, including touch vibration (Figure 5(d)), pressure (Figure 5(e)), magnetic force (Figure 5(f)), temperature (Figure 5(g)), and humidity (Figure 5(h)). In addition, this multisensory e-skin exhibited discernible current signal curves with excellent sensitivity, discriminability, and repeatability (Figure S15). These attributes indicate that this e-skin can easily distinguish different stimulation behaviors (such as touch, temperature, air flow, humidity, and even magnetic fields) by analyzing the magnitude and area of the produced electrical signal waveform.

Real human skin can actively sense varied information from the external environment. Notably, as shown in Figure 5(i), our e-skin can also simultaneously sense various stimuli, such as vibration, pressure, temperature, magnetic force, and humidity; it also demonstrates good repeatability and recognizability (Figure 5(i)). Through the observation of waveform signals (Figure S16), we can conveniently identify the intensity such as strain, pressure, temperature, and magnetic force. This is an important feature for an e-skin that is expected to rival human skin. By integrating the porcine gastrointestinal proteins into the proposed M-gel, the obtained e-skin can also clearly capture the astringent stimuli from citric acid (Figure 5(f)), which is difficult to achieve with other e-skins [41]. These features indicate that this e-skin based on M-gel is expected to help robots acquire a soft appearance and exhibit multiple perceptual capabilities for enhancing their uses in real-life settings.

## 3. Conclusions

In summary, we presented a facile and one-step method for introducing a thermionic source to directly convert biomass materials of BC hydrogel into ionic conductive M-gel materials. This M-gel possessed a designable multiscale structure consisting of a molecular-scale H-bonding topology network and a nanoscale fiber skeleton, which was attributed to the dynamic adjustability of the competitive relationship between molecularization and self-assembly. This endowed the M-gel with superior tunability in mechanical (2.5 and 15 MPa), optical (55.68% and 94.82%), and electronic (0 and 62.58 mS/cm) properties. Inspired by the distinct perceptual structure of the human tongue, we developed a skin-like multisensory e-skin device by constructing various sensing units (similar to the receptor cells of the tongue) in this M-gel. This e-skin demonstrated excellent sensing to pressure, touch, vibration, temperature, humidity, and magnetic force, indicating its broad application prospects in flexible electronics and artificial intelligence. The design method, controllable molecularization process, and scalable bionic e-skin design technique of the proposed ionogel are expected to result in the realization of smart robots with multiple perceptual abilities and the low-carbon sustainable development of flexible electronics.

## 4. Experimental Section

### 4.1. Materials and Chemicals

The BC hydrogel was purchased from Qihong Technology Co., Ltd. (Guilin, China). Nickel powder and citric acid were supplied by Macklin (Shanghai, China). 1-Methylimidazole (99%), 1-chlorobutane (99.8%), anhydrous calcium chloride (CaCl_2_), PEDOT: PSS, silver conductive paint, and CNTs were provided by Aladdin (Shanghai, China). Porcine gastrointestinal proteins were provided by Anhui Kuer Biological Engineering Co., Ltd. (Hefei, China).

### 4.2. Preparation of the Ionic Source

The ionic liquid 1-butyl-3-methylimidazolium chloride ([Bmim]Cl) was obtained using the method reported in a previous study [42]. The [Bmim]Cl used as the ionic source was prepared by stirring a mixture of 92.57 g 1-chlorobutane and 82.1 g 1-methylimidazole at 65°C for 30 min, followed by stirring at 85°C for 10 h under 1000 rpm and anhydrous calcium chloride protection. The stirred mixture subsequently was poured into 300 mL acetone. When the mixture cooled to room temperature, a crystal of [Bmim]Cl was formed. The white crystal of [Bmim]Cl was ultimately purified using rotary evaporation at 80°C for 1 h to remove the volatile impurities, resulting in a viscous, colorless, and *transparent ionic liquid.*

### 4.3. Preparation of the M-Gel

First, five times as much [Bmim]Cl of BC were weighed and filled onto the surface of a nonbubble BC hydrogel at 50°C. The BC hydrogel filled by an ionic source was then moved to an oven at 80°C and heat-treated for 0, 5, 10, 30, 50, and 70 min separately to obtain the M-gels. Through adjusting the processing time of thermionic sources, we can obtain the M-gels with different H_2_O and ion contents. The contents of H_2_O and ions source in M-gels can be calculated through the following process. First, we weighed the M-gel (including BC, H_2_O, and ion source) and recorded it as *M*_1_. Then, the M-gel was dried in oven at 110°C for 72 h to remove H_2_O, recording the weigh as *M*_2_. So, the H_2_O content (*C*_H2O_) in M-gel was calculated using the equation of *C*_H2O_ = 100% ∗(*M*_1_ − *M*_2_)/*M*_1_. Then, we placed the dried, water-free M-gel in distilled water to completely replace the ion source with water in M-gel, obtaining the hydrogel. We then dried the hydrogel in an oven at 110°C for 72 h, recording the weigh as *M*_3_. So, the ion source content (*C*_ion_) was calculated using the equation of *C*_ion_ = 100% ∗(*M*_2_ − *M*_3_)/*M*_1_. The properties of the M-gels treated over different lengths of molecularization time were compared.

### 4.4. Assembly of the Multisensory e-Skin

All stimulating receptors (made of nanomaterials, including CNTs, nickel powder, and PEDOT:PSS) were predried to remove moisture. The surface of the M-gel was covered with a shape-controllable mold and then preheated in an oven until the sticky M-gel was formed. The processed nanomaterials were then brushed on the M-gel as the stimulating receptors, followed by heat treatment in the oven, rendering it fit via M-gel H-bonding self-assembly. With an ear ball to blow off the active materials floating on the M-gel surface carefully, silver conductive paint was used to connect the stimulatory receptors, resulting in a multisensory e-skin.

### 4.5. Signal Test of the e-Skin

The multisensory e-skin was connected to a CHI760e electrochemical workstation (Chenhua Instruments, Shanghai, China). The current waveforms of biomimetic e-skin sensing touch, pressure, temperature, magnetic force, humidity, and airflow were assessed by measuring the amperometric I–t curve parameters with an initial potential of 0 V at room temperature.

## Data Availability

The data that support the findings of this study are available from the corresponding author upon reasonable request.

## Supplementary Materials

Supplementary Materials Figure S1: BC hydrogel is flexible and has the nanoscale fiber structure showed by the SEM image. Figure S2: preparation of M-gel by a simple thermionic treatment. Figure S3: light transmittance, T (%), of the M-gel during the different molecularization time. Figure S4: M-gel shows the designable crystallinity during different periods of thermionic molecularization. Figure S5: ionogel materials derived from other cellulose materials. Figure S6: the cyclic tensile tests and self-healing properties of M-gel. (a) Cyclic tensile tests of M-gel. (b) Self-healing behaviors of M-gel. Figure S7: optical images of the M-gel showing good adhesion to human wrists (a–c) and fingers (d–f). Figure S8: biocompatibility and corrosion testing. Figure S9: M-gel as the flexible conductor shows the good performance in the initial state of (a), bending state of (b), twisting state of (c), and folding state of (d). Figure S10: conductive stability of the M-gel in the air conditions with relative humidity of ≈45% for 30 days. Figure S11: conductive stability of the M-gel in folding process. Figure S12: adhesion properties of the M-gel material. Figure S13: schematic diagram of multisensory e-skin device sensing stimuli. Figure S14: structural integrity and stability testing of multisensory e-skin. (a) Finger rubbing. (b) Water immersion. Figure S15: current waveforms of the biomimetic e-skin sensing the vibration of (a), pressure of (b), magnetic force of (c), temperature of (d), humidity of (e), and airflow of (f), respectively. Figure S16: current waveforms of the e-skin sensing the changes in bending (a), pressure (b), temperature (c), and magnetic force (d).
