# Supplementary material for: A Scalable Bacterial Cellulose Ionogel for Multisensory Electronic Skin
Source: Research (Wash D C). 2022 Jun 2;2022:9814767. doi: 10.34133/2022/9814767 (PMC9188022; doi:10.34133/2022/9814767)
Supplement: Supplementary Materials — Figure S1: BC hydrogel is flexible and has the nanoscale fiber structure showed by the SEM image. Figure S2: preparation of M-gel by a simple thermionic treatment. Figure S3: light transmittance, T (%), of the M-gel during the different molecularization time. Figure S4: M-gel shows the designable crystallinity during different periods of thermionic molecularization. Figure S5: ionogel materials derived from other cellulose materials. Figure S6: the cyclic tensile tests and self-healing properties of M-gel. (a) Cyclic tensile tests of M-gel. (b) Self-healing behaviors of M-gel. Figure S7: optical images of the M-gel showing good adhesion to human wrists (a–c) and fingers (d–f). Figure S8: biocompatibility and corrosion testing. Figure S9: M-gel as the flexible conductor shows the good performance in the initial state of (a), bending state of (b), twisting state of (c), and folding state of (d). Figure S10: conductive stability of the M-gel in the air conditions with relative humidity of ≈45% for 30 days. Figure S11: conductive stability of the M-gel in folding process. Figure S12: adhesion properties of the M-gel material. Figure S13: schematic diagram of multisensory e-skin device sensing stimuli. Figure S14: structural integrity and stability testing of multisensory e-skin. (a) Finger rubbing. (b) Water immersion. Figure S15: current waveforms of the biomimetic e-skin sensing the vibration of (a), pressure of (b), magnetic force of (c), temperature of (d), humidity of (e), and airflow of (f), respectively. Figure S16: current waveforms of the e-skin sensing the changes in bending (a), pressure (b), temperature (c), and magnetic force (d). [file 9814767.f1.doc]

**Supporting information**

**A Scalable Bacterial Cellulose Ionogel for Multisensory Electronic Skin**

Geyuan Jiang^1^, Gang Wang^1^, Ying Zhu^2^, Wanke Cheng^2^, Kaiyue Cao^2^, Guangwen Xu^1^, Dawei Zhao^1,2,3^*, Haipeng Yu^2^*

1. Key Laboratory on Resources Chemicals and Materials of Ministry of Education, Shenyang University of Chemical Technology, Shenyang 110142, P. R. China
2. Key Laboratory of Bio-based Material Science and Technology of Ministry of Education, Northeast Forestry University, Harbin 150040, P. R. China
3. Tianjin Key Laboratory of Pulp and Paper, Tianjin University of Science and Technology, Tianjin 300457, P. R. China

**I. Supplementary Methods**

*Characterization.* The SEM microstructure of sample was characterized by the JSM-7500F microscope (Hitachi, Tokyo, Japan) at an operating voltage of 10 kV. The micro-morphology of samples was investigated by the AFM of the RTESP-300 (Bruker, USA) at the tapping mode. The FTIR spectra of sample were obtained by a Nicolet 6700 FTIR instrument (Thermo Fisher Scientific Inc., Waltham, MA, USA). All spectra were measured in ATR mode, with data recorded in the range of 600–4000 cm^-1^ over 32 scans with a resolution of 4 cm^-1^. The XRD patterns of sample were measured by a D/max 2200 X-ray diffractometer (Rigaku, Tokyo, Japan) equipped with Ni-filtered Cu-Kα radiation (λ = 0.154 nm). The samples were scanned within 5–90° 2θ at 40 kV and 30 mA with a scanning rate of 2° min^-1^. The small angle X-ray scattering (SAXS) of samples was measured using the SAXSess mc2 instrument with Cu Kα X-ray radiation and a wavelength of 0.154 nm (Anton Paar, Graz, Austria). The sample-to-detector distance was 2658.5 mm and exposure time was 5 min.

*Ionic conductivity measurement of the M-gel.* For ionic conductivity, the M-gels were tested like thin film. First, two nickel sheets served as conductors were sandwiched on both ends of a M-gel, where the overlapping length of the conductor was approximately 1 cm. Two copper wires were attached to both another ends of conductors. Then the M-gel system was connected to a CHI760e electrochemical workstation (Chenhua Instruments, Shanghai, China) and measured the AC impedance spectrum (AC amplitude of 1 mV, frequencies between 100 mHz and 1 MHz). The bulk resistance of the M-gel can be measured by EIS. The ionic conductivity of M-gel was calculated by combining the bulk resistance of the M-gel, the distance between the two electrodes and the effective cross-sectional area of the M-gel, which was equivalent to the end area of the electrode sheet.

*Calculate the DM of M-gel at different molecularization periods.* The light transmittance value (T %) of M-gel at different molecularization periods was tested by the JDY-100 digital hazemeter (Jindouyun Precision Instrument Co., LTD, Jiangsu, China) at wavelength of 550 nm. The DM of M-gel is calculated by the following equation:

*DM = (T_x_-T_BC hydrogel_) × (100/(T_M-gel-70_ ‒ T_BC hydrogel_)) × 100%*

where *T_X_* is the T % of M-gel treated by different thermionic molecularization time, *T_BC hydrogel_* is the T % of original BC hydrogel film, and *T_M-gel-70_* is the T % of the M-gel by complete molecularization (thermionic treatment for 70 min).

**II. Supplementary Figures**

**
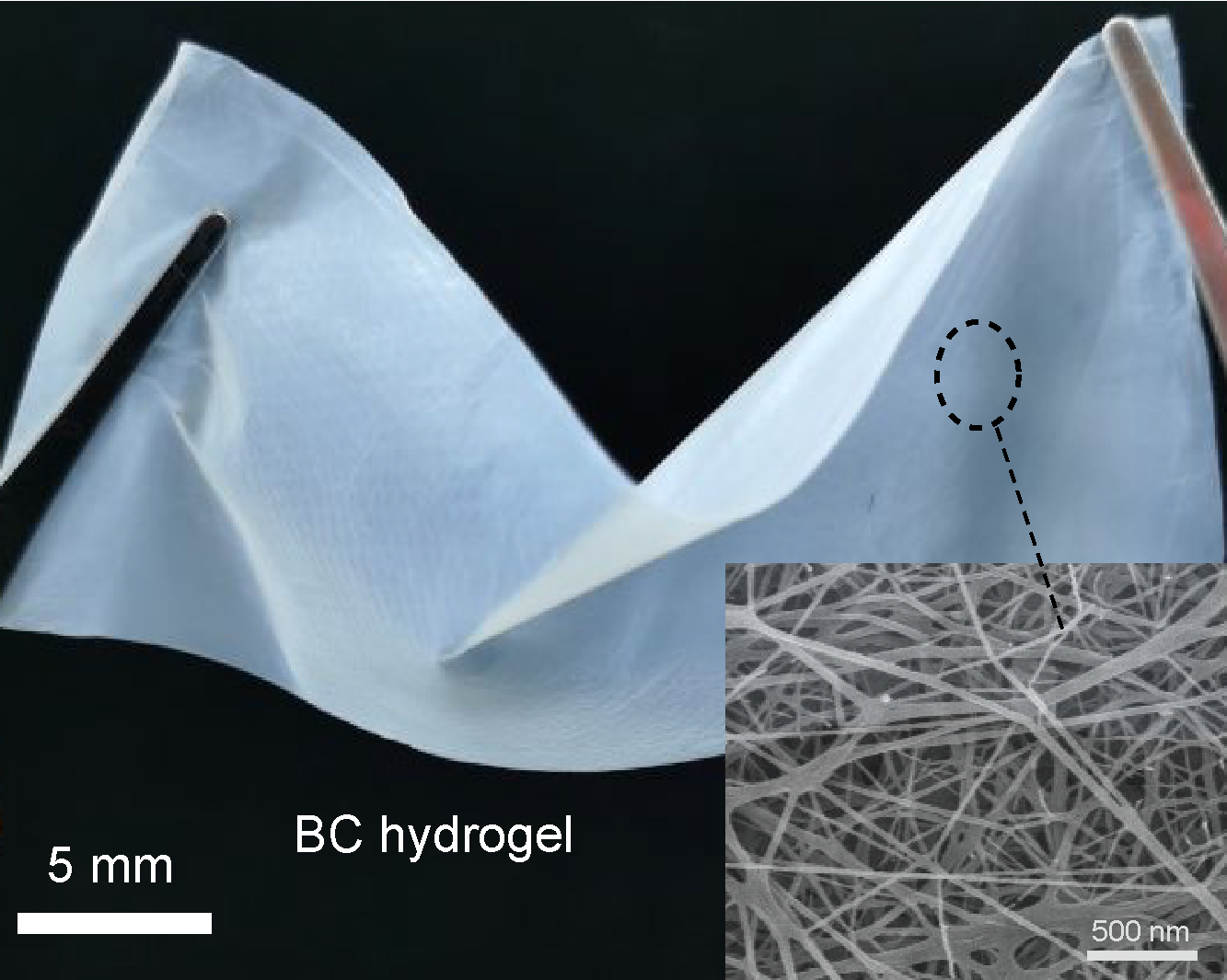
**

FIGURE S1: BC hydrogel is flexible and has the nanoscale fiber structure showed by the SEM image.


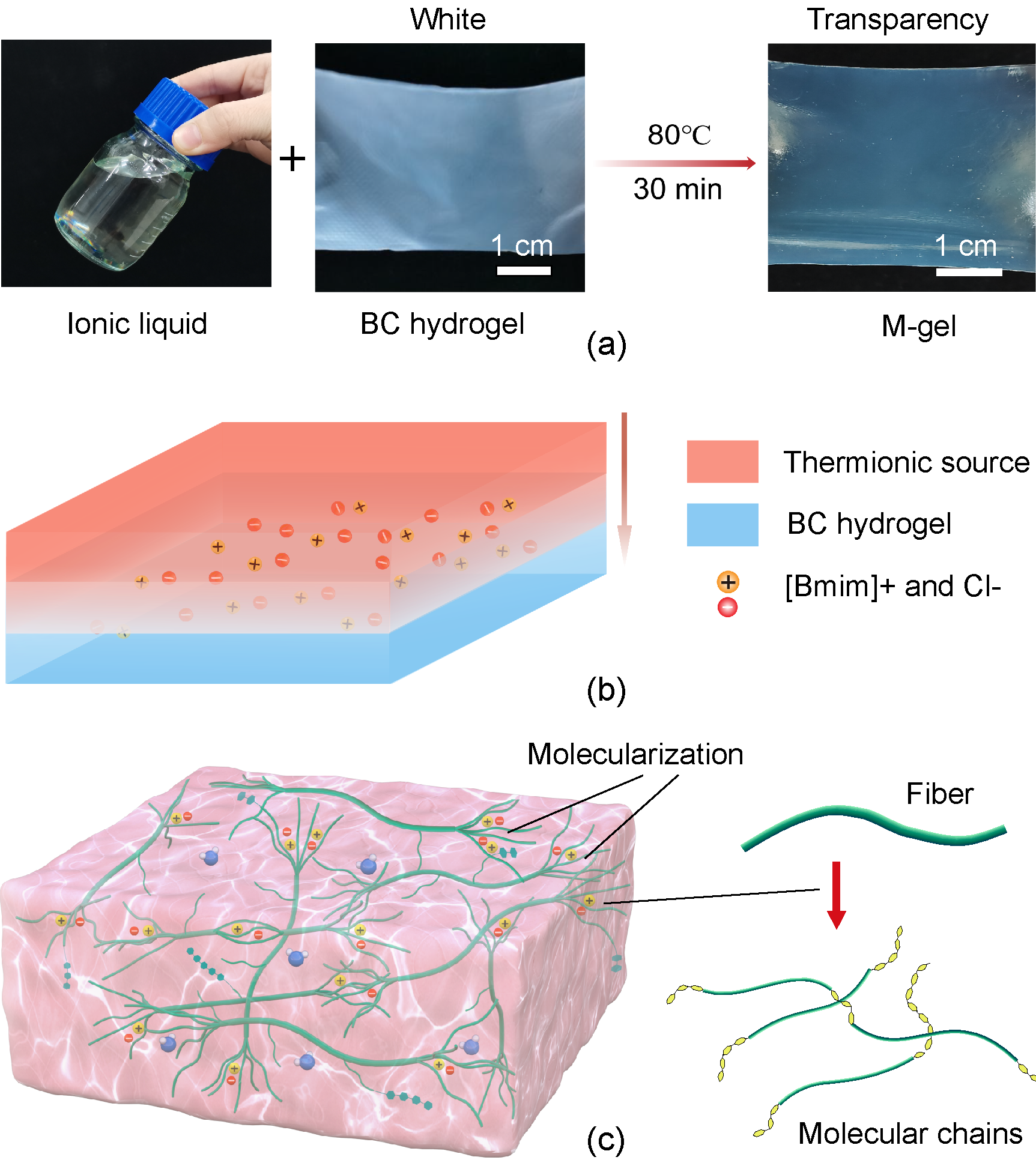


FIGURE S2: Preparation of M-gel by a simple thermionic treatment. (a) Optical images of ionic liquid, BC hydrogel, and M-gel (treated by thermionic source at 80 °C for 30 min). (b) schematic of thermal diffusion of anion-cation in BC hydrogel. (c) Molecularization process of BC fiber converted into cellulose molecular chains.


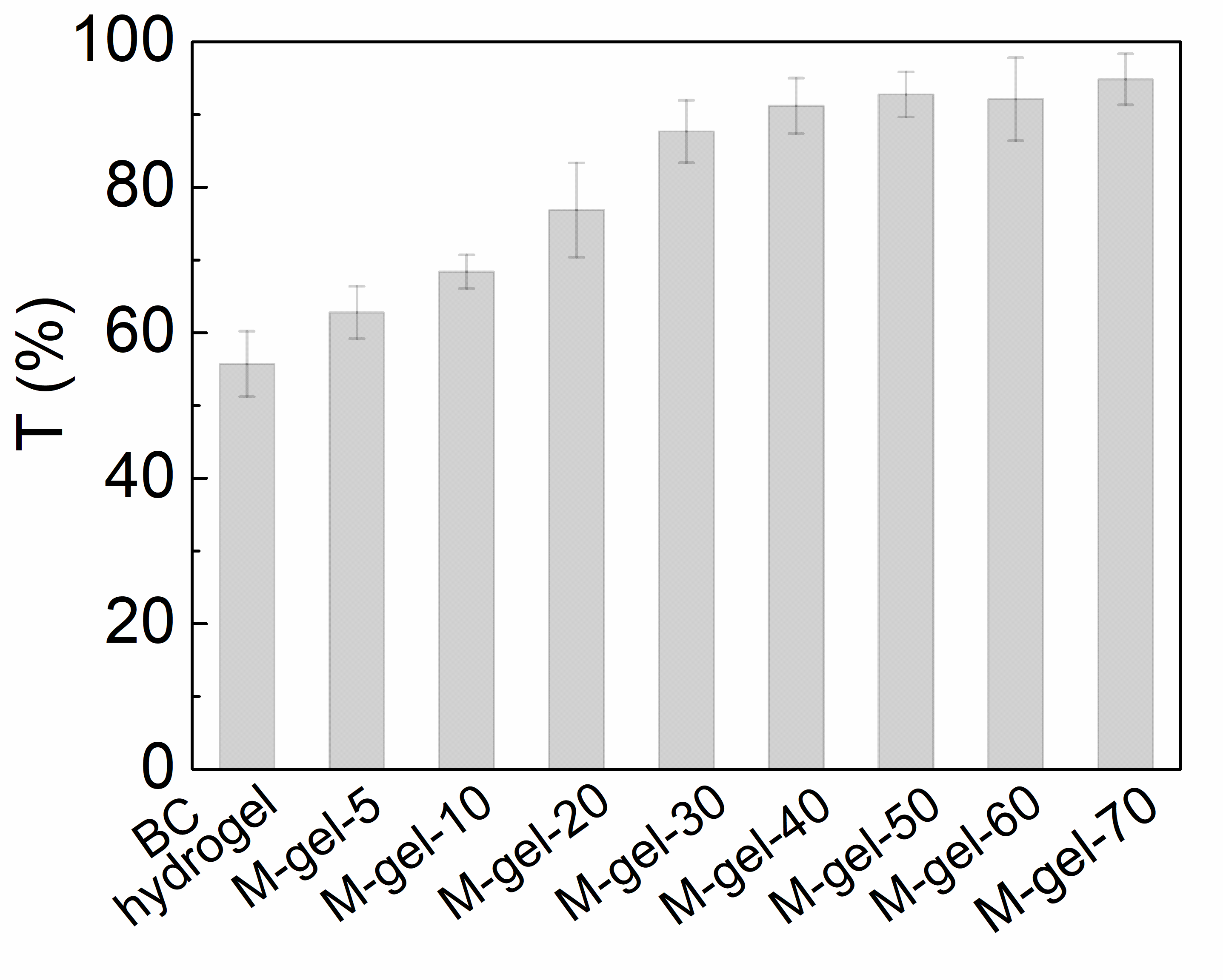


FIGURE S3: Light transmittance, T (%), of the M-gel during the different molecularization time.


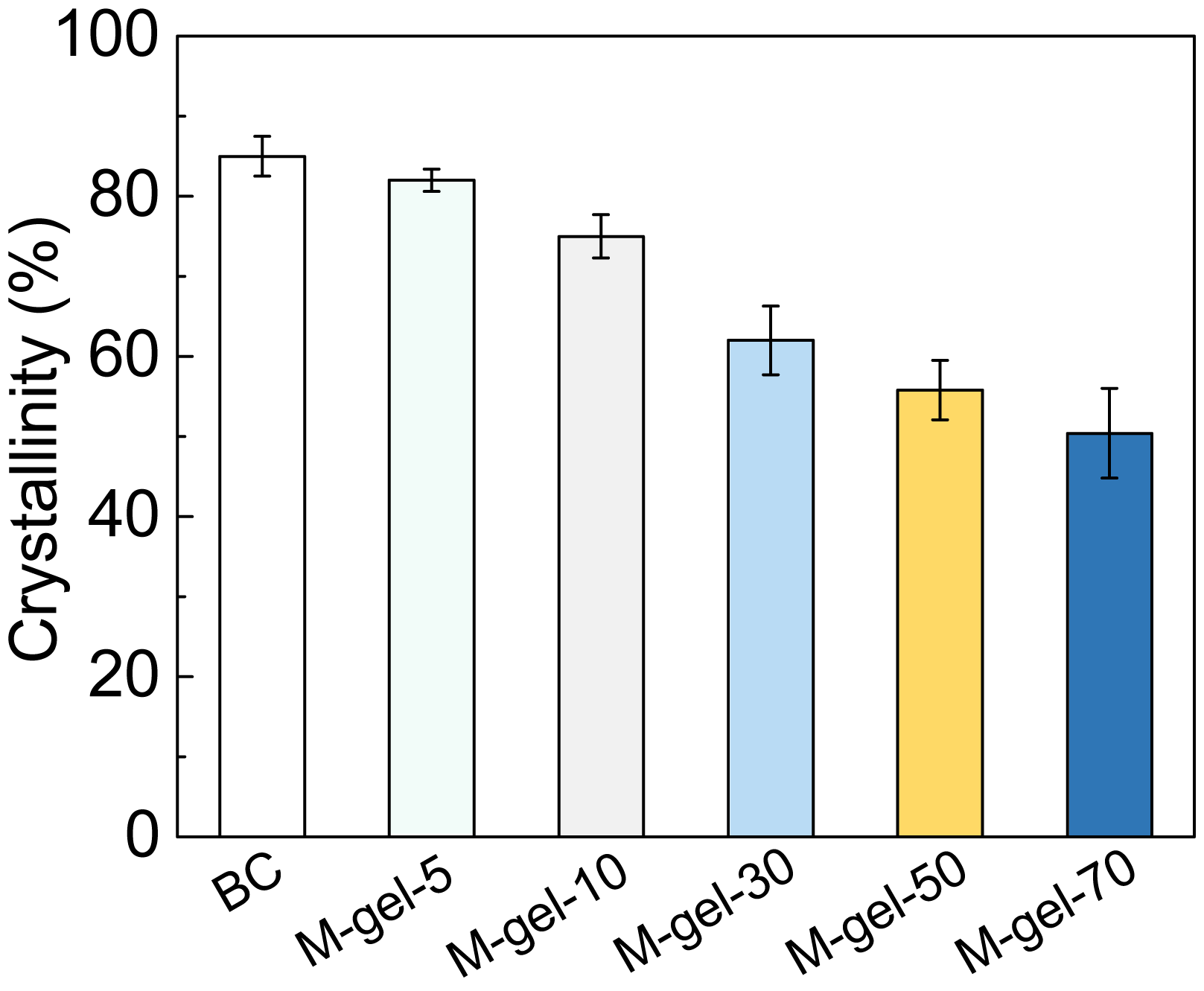


FIGURE S4: M-gel shows the designable crystallinity during different periods of thermionic molecularization.


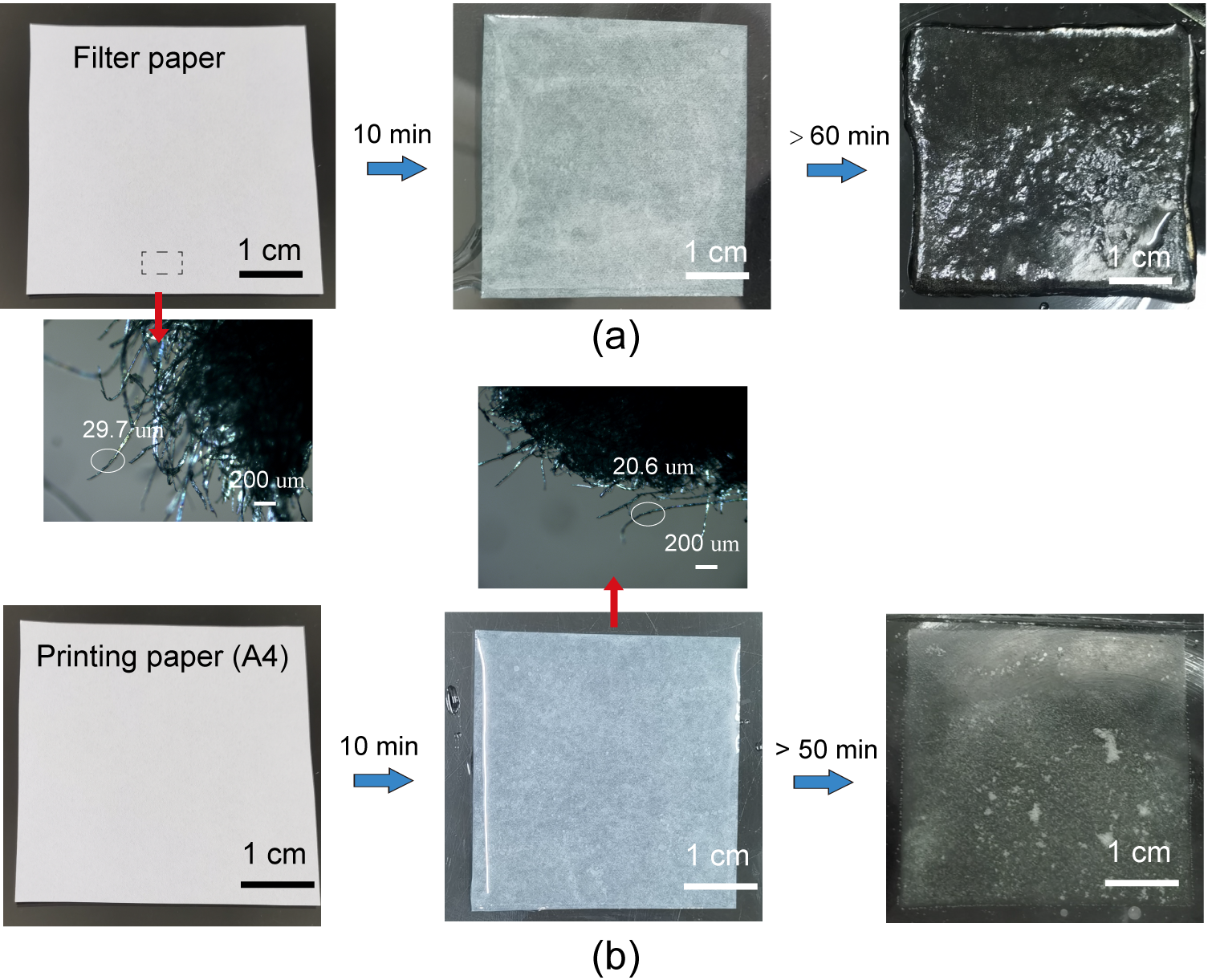


FIGURE S5: Ionogel materials derived from other cellulose materials (a) Molecularization process of filter paper for ionogel material. (b) Molecularization process of printing paper (A4) for ionogel material.


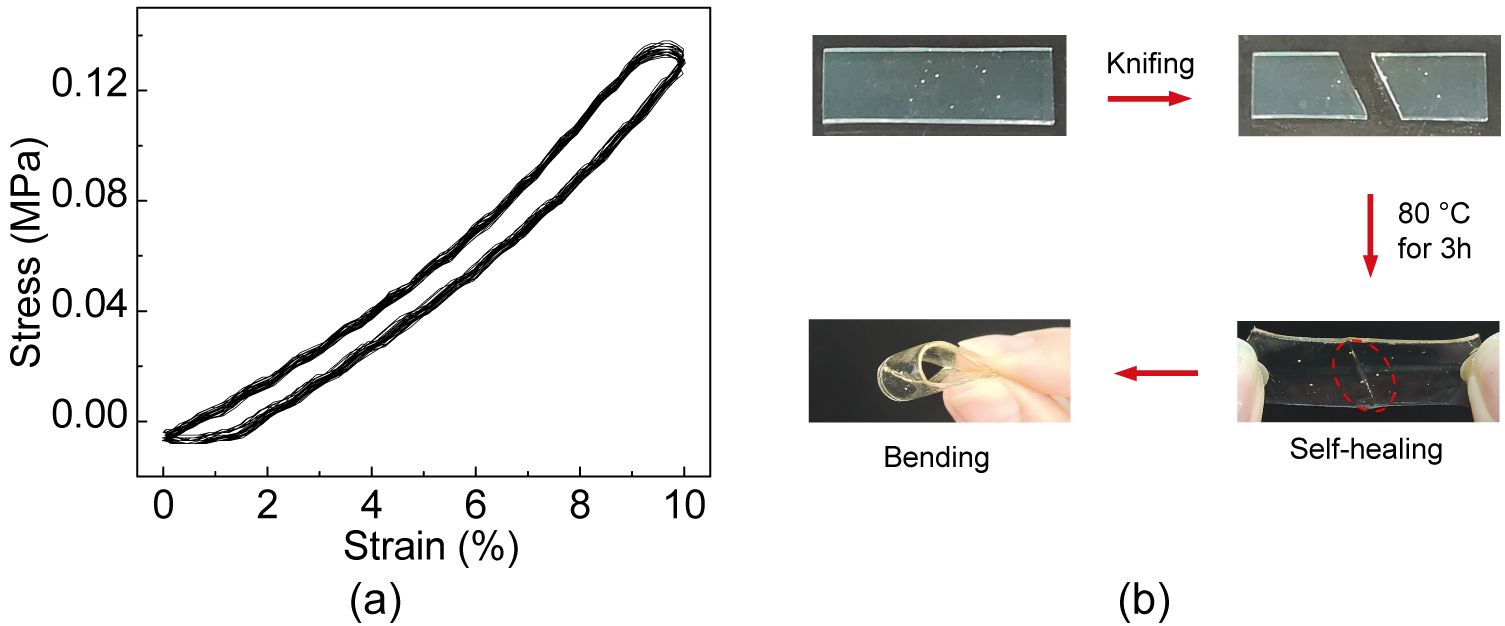


FIGURE S6: The cyclic tensile tests and self-healing properties of M-gel. (a) Cyclic tensile tests of M-gel. (b) Self-healing behaviors of M-gel.

**
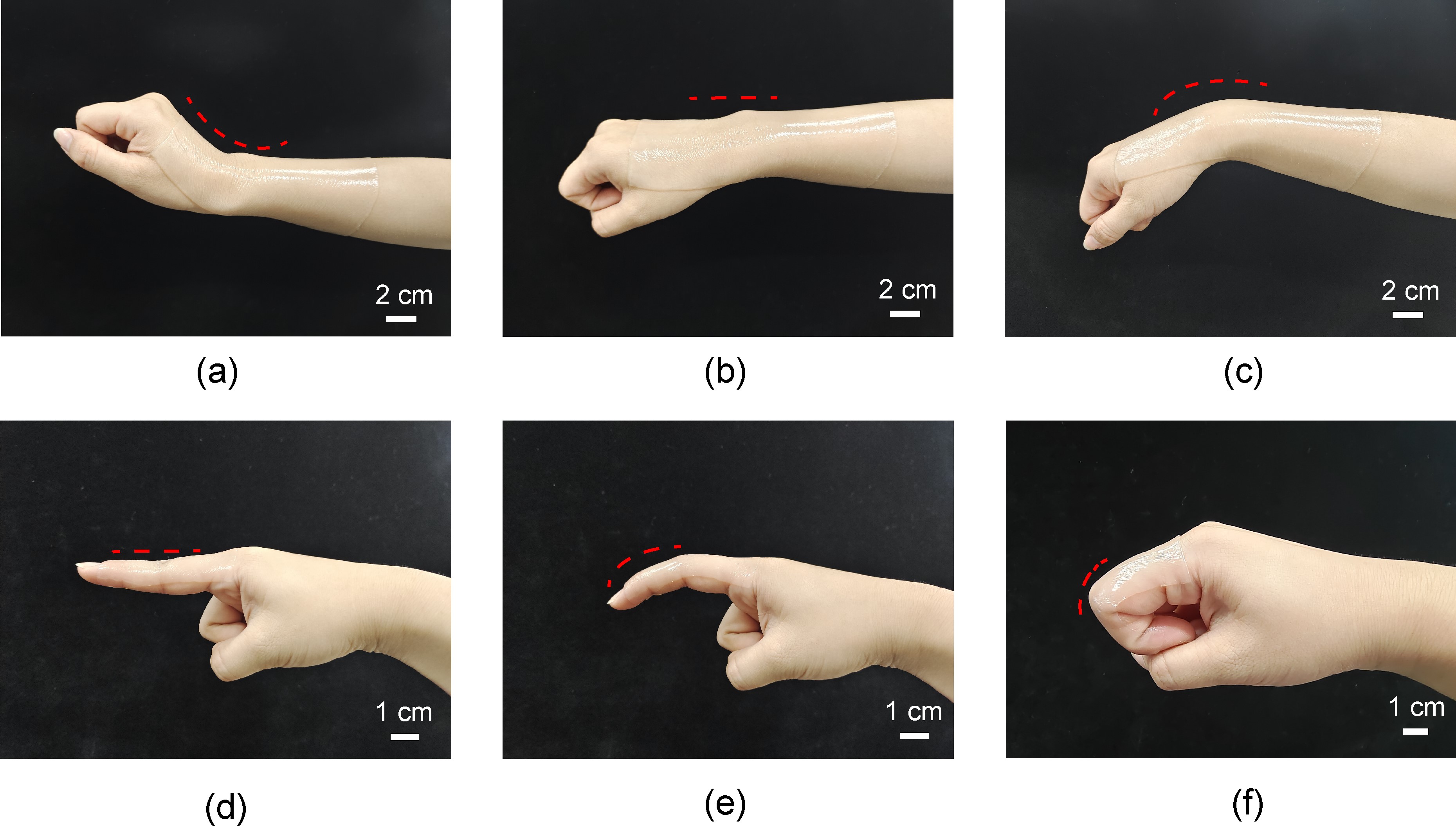
**

FIGURE S7: Optical images of the M-gel showing good adhesion to human wrists (a)-(c) and fingers (d)-(f).

**
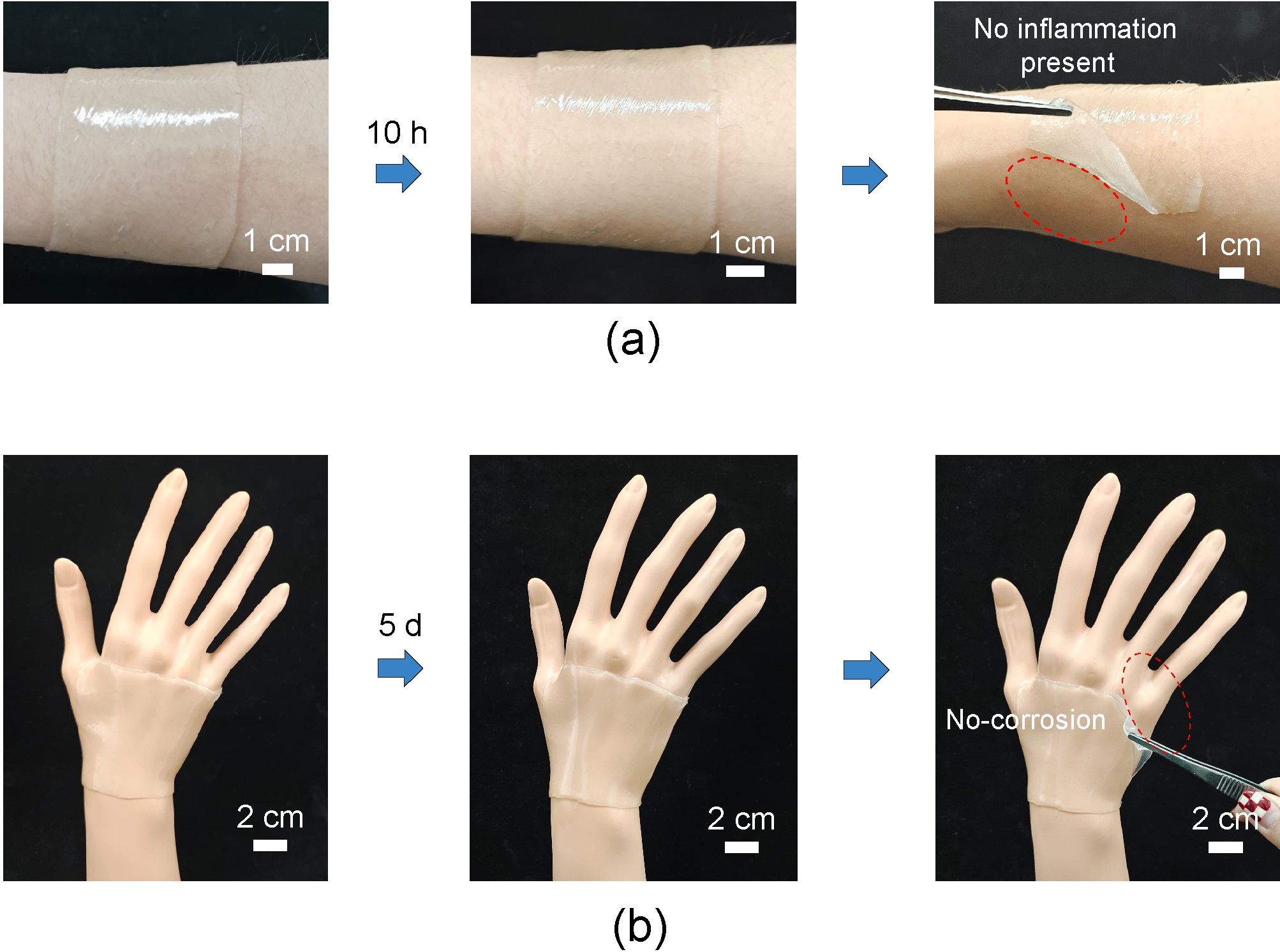
**

FIGURE S8: Biocompatibility and corrosion testing. (a) Biocompatibility test of M-gel in human body. (b) Corrosion test of M-gel on artificial hand (made up of polyvinyl chloride resin).


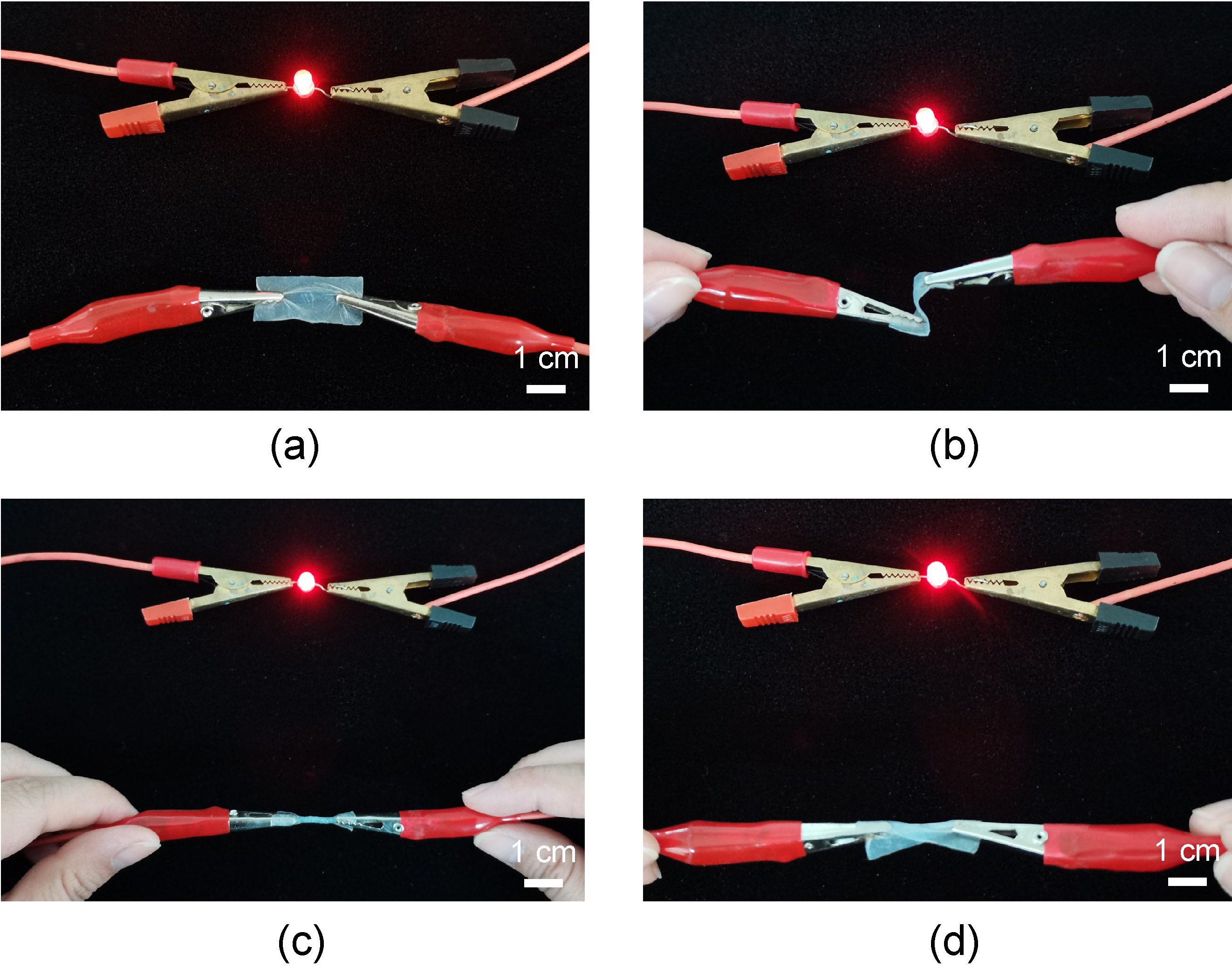


FIGURE S9: M-gel as the flexible conductor shows the good performance in the initial state of (a), bending state of (b), twisting state of (c), and folding state of (d).


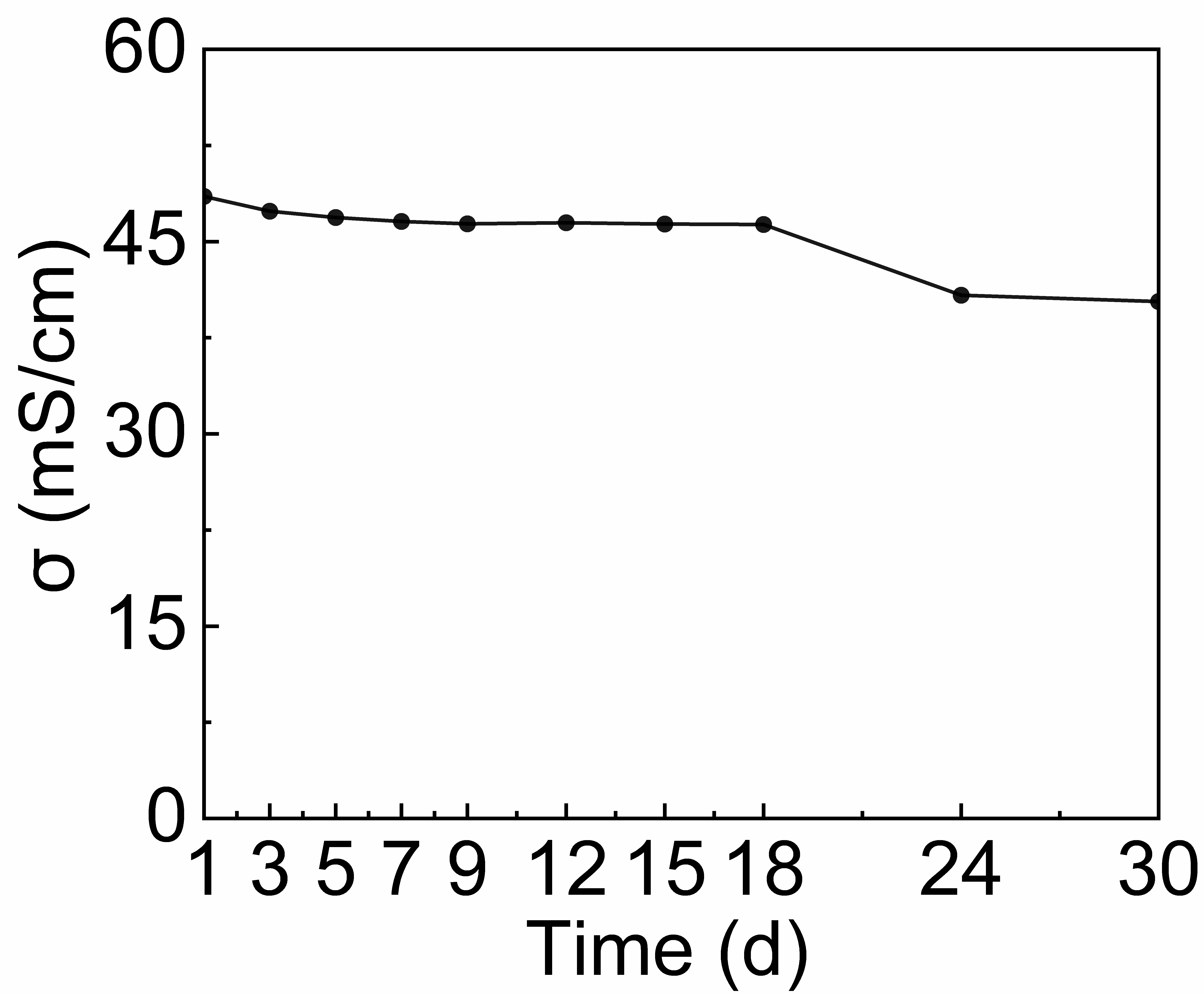


FIGURE S10: Conductive stability of the M-gel in the air conditions with relative humidity of ≈ 45% for 30 days.


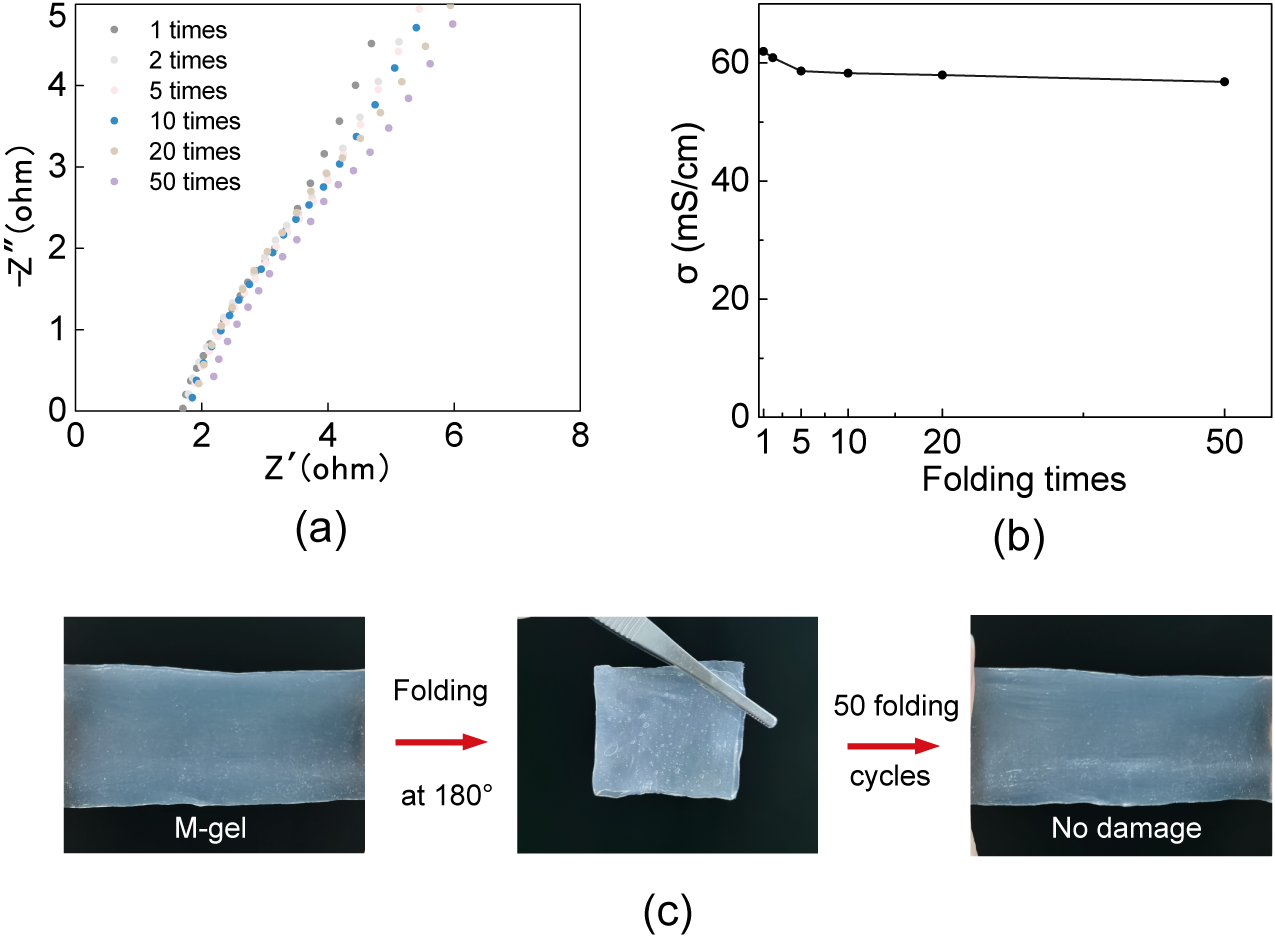


FIGURE S11: Conductive stability of the M-gel in folding process. (a) EIS curves of M-gel with the folding angle of 180° after different folding cycles. (b) Conductivity of M-gel under different folding cycles. The folding angle is 180°. (c) Optical images of M-gel in the initial state, folded state, and after 50 cycles of folding.


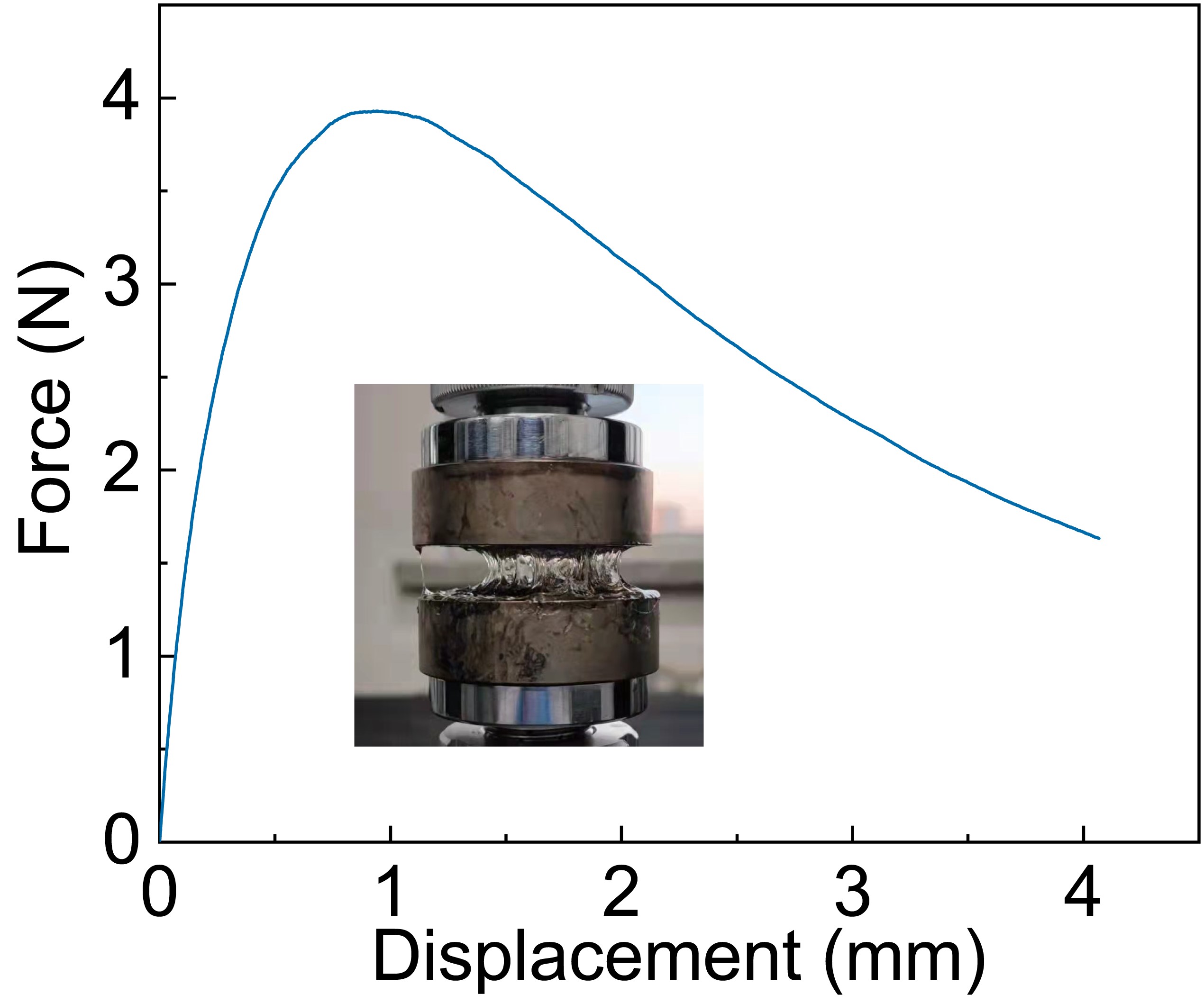


FIGURE S12: Adhesion properties of the M-gel material.


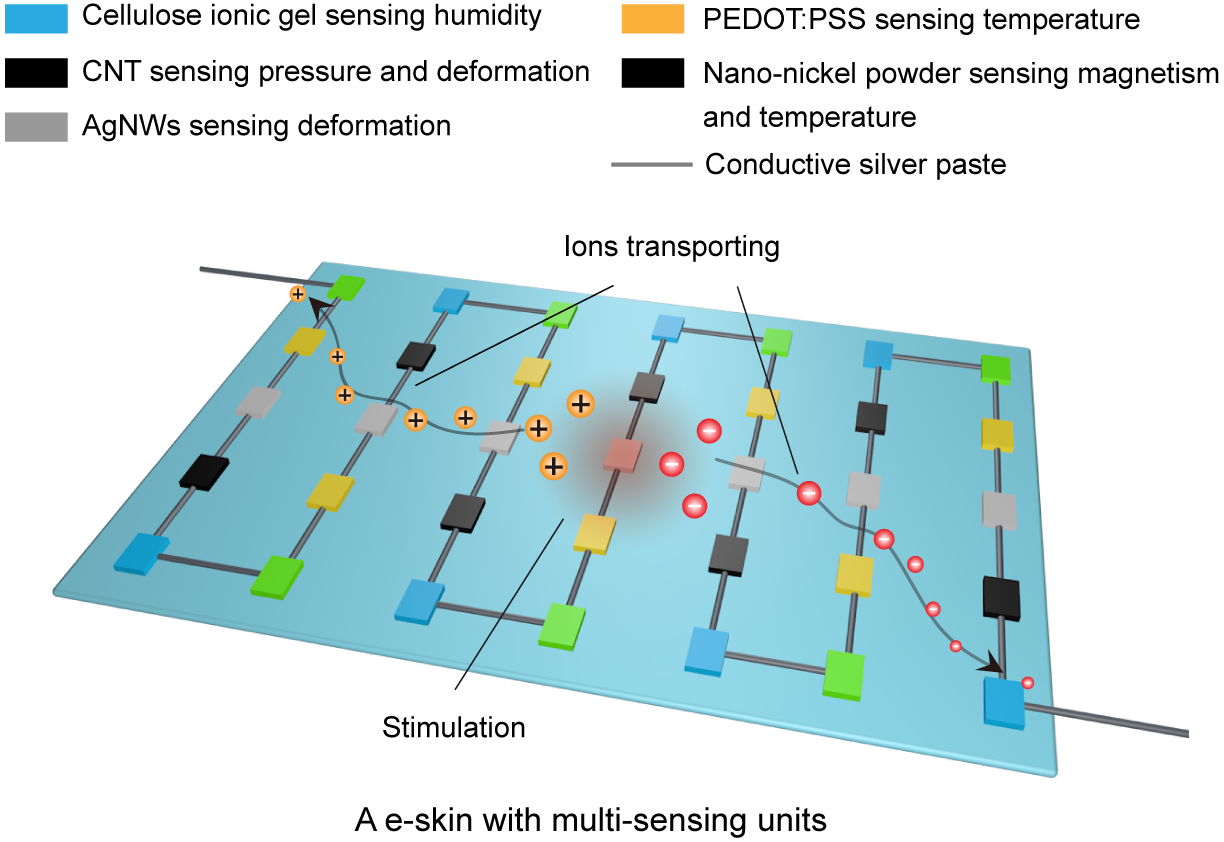


FIGURE S13: Schematic diagram of multisensory e-skin device sensing stimuli.


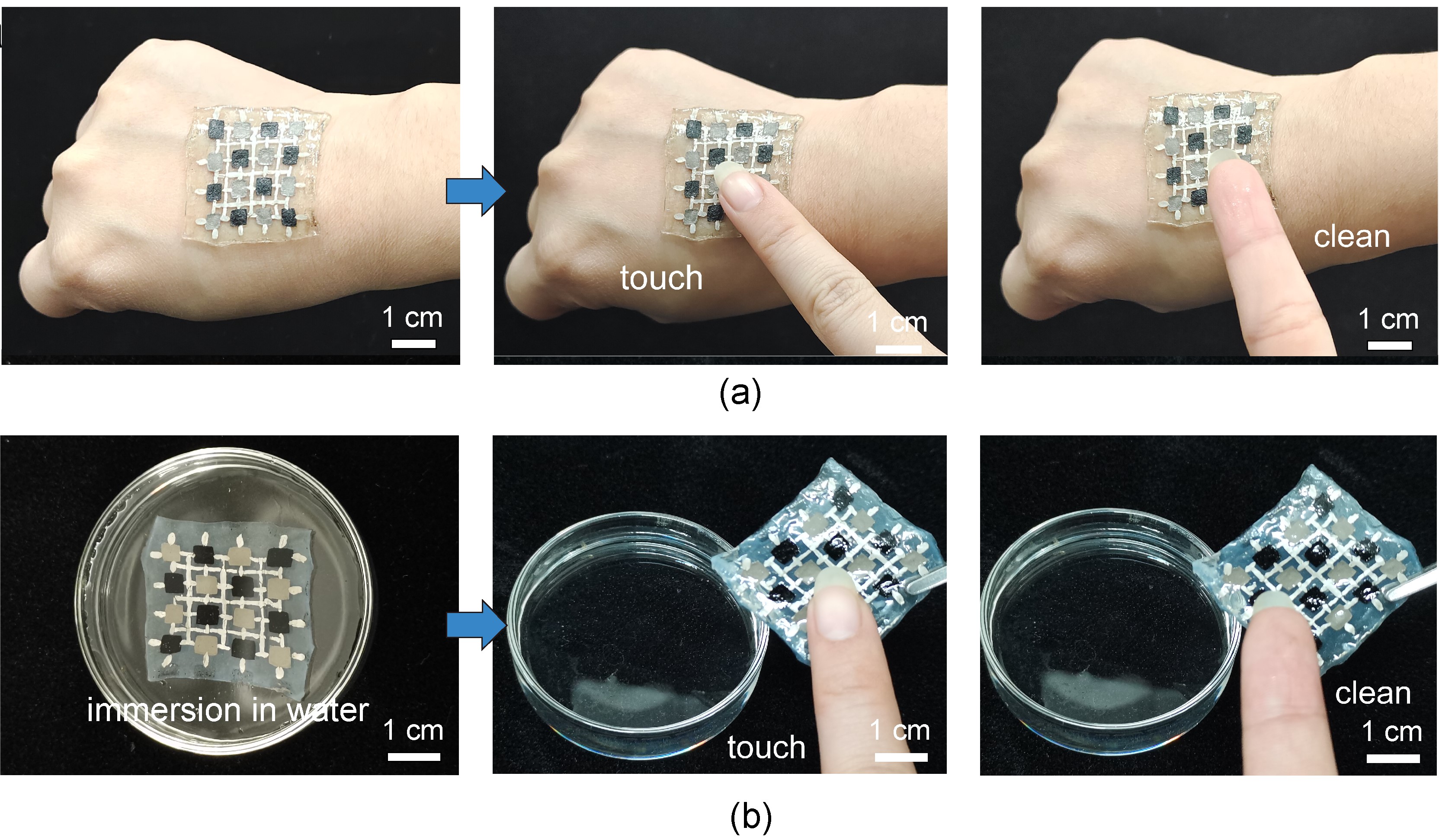


FIGURE S14: Structural integrity and stability testing of multisensory e-skin. (a) Finger rubbing. (b) Water immersion.


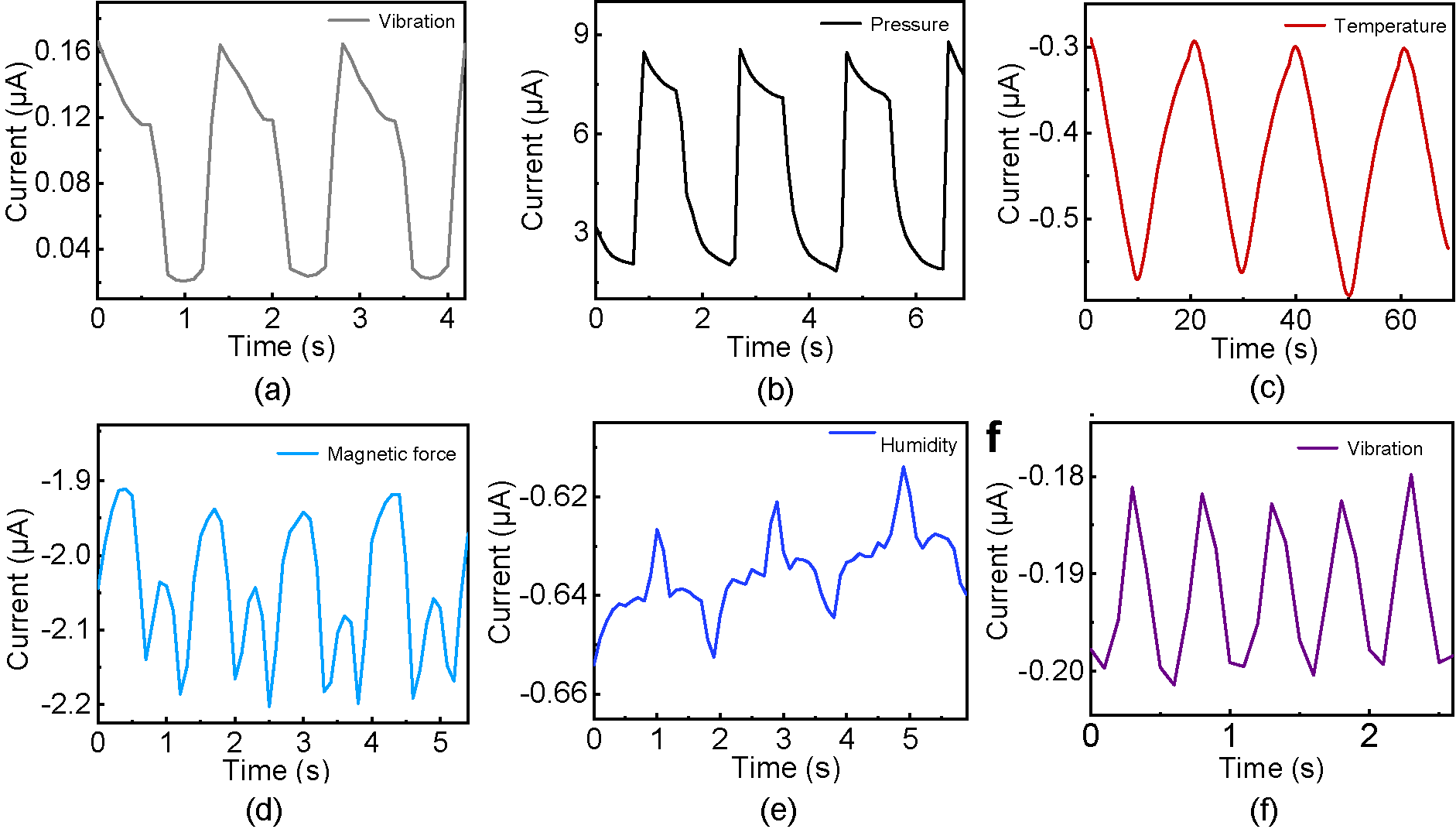


FIGURE S15: Current waveforms of the biomimetic e-skin sensing the vibration of (a), pressure of (b), magnetic force of (c), temperature of (d), humidity of (e), and airflow of (f), respectively.


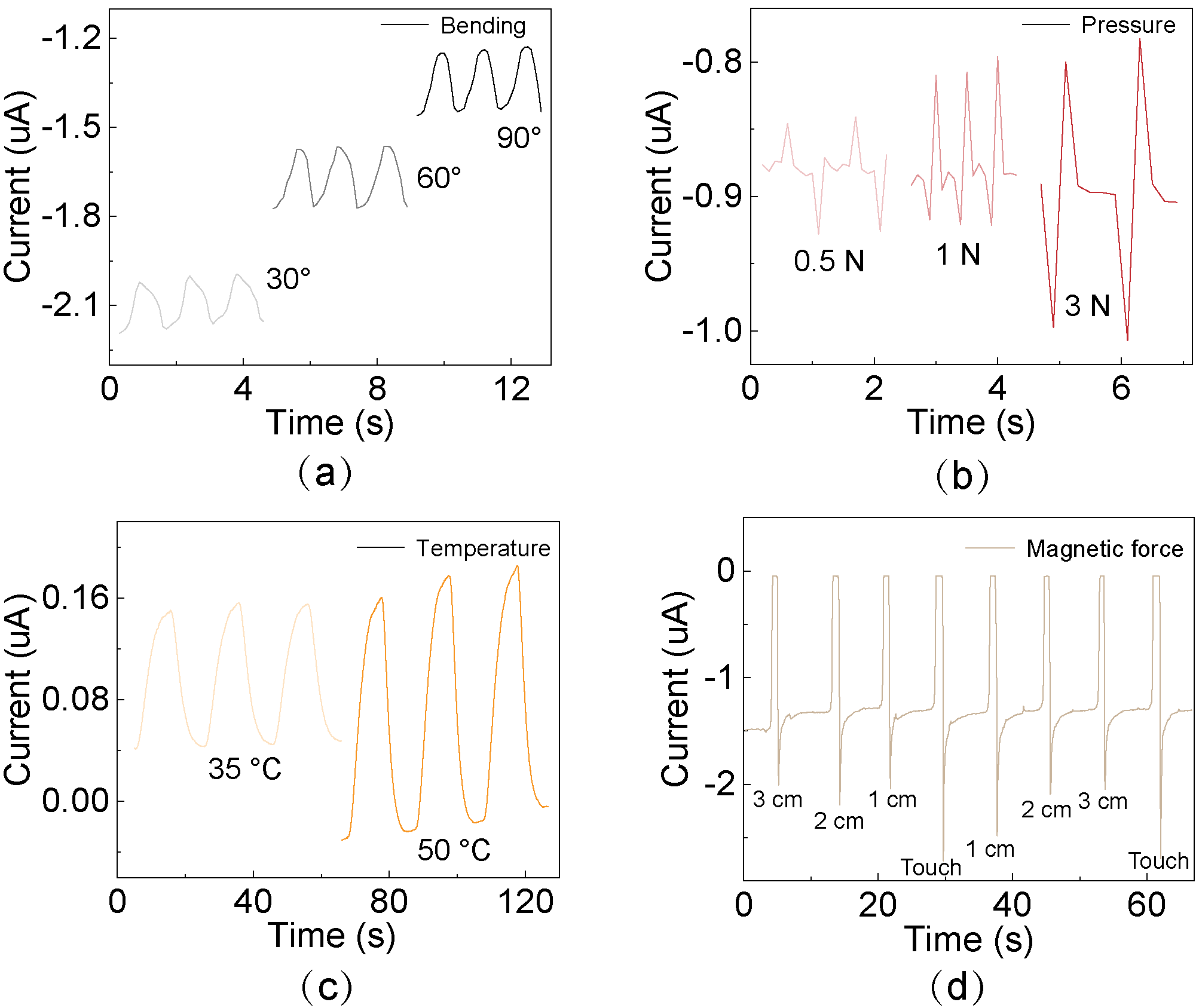


FIGURE S16: Current waveforms of the e-skin sensing the changes in bending (a), pressure (b), temperature (c), and magnetic force (d).
